# Participation Outcomes One Year After Aneurysmal Subarachnoid Hemorrhage: Associations with Cognition, Coping, and Psychological Distress

**DOI:** 10.3390/neurosci6040128

**Published:** 2025-12-10

**Authors:** Angelka Pešterac-Kujundžić, Una Nedeljković, Ivana Sretenović, Aleksandar Milosavljević, Dragoslav Nestorovic, Ivan Vukašinović, Vojislav Bogosavljević

**Affiliations:** 1 College of Health Sciences, Academy of Applied Studies Belgrade, Cara Dušana 254, 11000 Belgrade, Serbia; 2 Faculty of Medicine, University of Belgrade, 11000 Belgrade, Serbia; 3 Center for Physical Medicine and Rehabilitation, University Clinical Center of Serbia, 11000 Belgrade, Serbia; 4 Faculty of Special Education and Rehabilitation, University of Belgrade, 11000 Belgrade, Serbia; 5 Clinic for Neurosurgery, University Clinical Center Kragujevac, 34000 Kragujevac, Serbia; 6 Center for Radiology and MRI, University Clinical Center of Serbia, 11000 Belgrade, Serbia; 7 Clinic for Neurosurgery, University Clinical Center of Serbia, 11000 Belgrade, Serbia

**Keywords:** subarachnoid hemorrhage, social participation, psychological distress, coping behavior, cognition

## Abstract

This study evaluated participation outcomes one year after aneurysmal subarachnoid hemorrhage (aSAH) compared with matched healthy controls and identified factors associated with participation within the patient group. Forty aSAH survivors and seventy-five controls were assessed 12–14 months post-ictus. Participation was measured with the Utrecht Scale for Evaluation of Rehabilitation–Participation (USER-P), psychological distress with the Hospital Anxiety and Depression Scale (HADS), coping with the Brief COPE, and cognition with the Montreal Cognitive Assessment (MoCA). Compared with controls, patients reported greater participation restrictions (82 vs. 100, *p* < 0.001), lower frequency (35 vs. 51, *p* < 0.001), and reduced satisfaction (65 vs. 75, *p* < 0.001). Anxiety, depression, and avoidant coping independently predicted restrictions (adjusted R^2^ = 0.48), while satisfaction was predicted by employment, fewer depressive symptoms, and less avoidant coping (adjusted R^2^ = 0.52). Lower MoCA scores predicted reduced participation frequency (*p* = 0.032), and patients with cognitive impairment showed significantly greater restrictions and lower satisfaction. One year after aSAH, survivors experience substantial participation limitations associated with psychological distress, maladaptive coping, and cognitive deficits. These results underscore the importance of cognitive and psychological rehabilitation to enhance long-term participation and social reintegration after aSAH.

## 1. Introduction

Aneurysmal subarachnoid hemorrhage (aSAH) is a sudden, life-threatening cerebrovascular event that predominantly affects individuals in the working-age population [1]. Despite significant advancements in the overall management of aSAH [2], recovery is often complex and extends beyond resolving the initial neurological insult, as psychological functioning has been increasingly recognized as a key aspect of long-term outcome [3].

Although many survivors achieve good neurological outcomes, persistent fatigue, anxiety, depression, and cognitive dysfunction are common [4,5,6]. Mood disturbances are among the most frequent psychological consequences, affecting up to 40–60% of survivors in the first year post-ictus [4,7,8]. Ackermark et al. [9] reported increasing rates of depression over time, while anxiety remained elevated in about half of patients, a finding confirmed by von Vogelsang et al. [10] during a two-year follow-up. These disturbances are strongly linked to poorer functional outcomes, including reduced quality of life, greater disability, and lower return-to-work rates [8,11].

In addition to mood disturbances, cognitive impairments are frequently reported after aSAH, even in patients with good neurological recovery. Deficits in memory, executive functions, and language are the most common and may persist long-term, adversely affecting independence and quality of life [12]. Importantly, such impairments have been shown to significantly influence participation, with lower cognitive performance and subjective complaints strongly associated with greater restrictions and dissatisfaction in social, domestic, and occupational roles [13,14,15].

Coping strategies represent the ways individuals manage stressors after major health events such as aSAH, shaping their emotional responses and interaction with the environment. Passive styles (e.g., disengagement, self-blame) are linked to greater depression, anxiety, and lower quality of life [9,16], whereas active, problem-focused coping predicts better psychological adjustment [17,18].

Cognitive, emotional, and behavioral responses following aSAH influence psychological well-being and the resumption of daily roles. Participation is defined as engagement in meaningful activities at home, in the community, or at work and is increasingly recognized as a key dimension of recovery after aSAH [13]. Despite independence in basic self-care, many survivors struggle to reintegrate into social, domestic, or occupational roles. Participation restrictions and dissatisfaction are common even among ADL-independent patients, particularly in housekeeping, outdoor activities, and employment [13,14], and are closely associated with cognitive complaints and mood symptoms [13,19].

Difficulties in participation are among the most disabling sequelae after this condition, yet the interplay between cognitive performance, emotional status, and coping behavior remains insufficiently understood. A better understanding of these interrelations is essential to identify individuals at risk of poor psychosocial recovery and to guide targeted rehabilitation strategies. To comprehensively assess real-life functioning after aSAH, participation in the present study was measured using the Utrecht Scale for Evaluation of Rehabilitation–Participation (USER-P), an instrument conceptually grounded in the participation com-ponent of the International Classification of Functioning, Disability and Health (ICF). The USER-P distinguishes between frequency, restrictions, and satisfaction with daily activities, thus capturing both objective and subjective aspects of participation [13].

This study primarily aimed to evaluate participation outcomes in patients one year after aneurysmal subarachnoid hemorrhage, in comparison with matched healthy controls. A secondary objective was to identify demographic, clinical, psychological, cognitive, and coping-related factors associated with participation within the patient group.

## 2. Material and Methods

### 2.1. Study Design and Setting

This prospective observational cohort study with an externally recruited matched healthy control group was conducted at the Clinic for Neurosurgery, University Clinical Center of Serbia, Belgrade, between November 2022 and November 2024. All patients were followed for 12–14 months after the ictus. The study was approved by the Institutional Ethics Committee (No. 1039/3, 26 October 2022), and all participants provided written informed consent.

### 2.2. Participants

**Figure 1:**
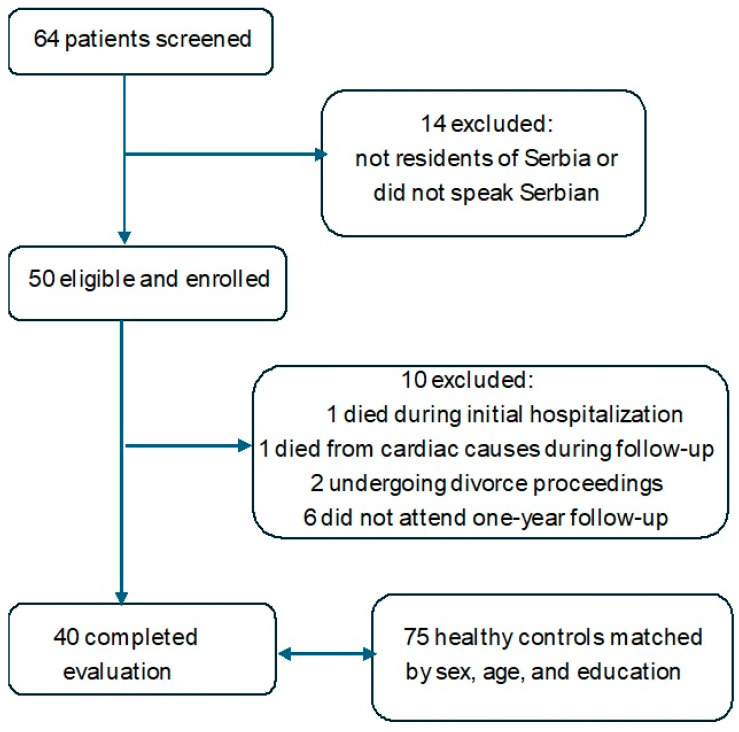
Flowchart of the inclusion process. Legend: Flow of participants from initial screening to final inclusion in the study.

Patients were consecutively enrolled after endovascular embolization if they presented with Hunt and Hess grade I–II on admission, after which additional inclusion and exclusion criteria were applied. Inclusion criteria were age ≥ 18 years, fluency in Serbian, residence in Serbia. Exclusion criteria were traumatic or non-aneurysmal SAH, severe comorbidities (e.g., active malignancy, NYHA III–IV heart failure) previous cerebrovascular events, or residence outside Serbia. During the study period 64 patients with H & H Grade I and II underwent endovascular treatment for aSAH, but only 50 were eligible for inclusion in the study. Of 50 eligible patients, 10 were excluded (two deaths—one during initial hospitalization and one during follow-up, two undergoing divorce proceedings, and six lost to follow-up), leaving 40 for analysis. The selection process and matching with controls are shown in Figure 1.

Most patients were physically independent at discharge. Two required post-acute inpatient rehabilitation due to residual motor deficits: one had mild hemiparesis (Modified Rankin Scale [mRS] = 2), which improved after rehabilitation, while another was fully dependent at discharge (mRS 4) but regained independent ambulation by the one-year follow-up.

The control group consisted of neurologically healthy adults recruited from the patients’ social environment and the local community. Each patient was matched by sex, age, and education level with one or two controls (*n* = 75 in total). Inclusion criteria for controls were fluency in Serbian, and absence of neurological or psychiatric disease. Exclusion criteria included a history of cerebrovascular events, malignancy, NYHA class III–IV cardiovascular disease, or severe physical disability.

### 2.3. Measures

During hospital admission, demographic and clinical data were extracted from medical records and included age, sex, level of education, aneurysm location, Hunt and Hess (HH) grade at admission, and length of hospital stay (LoS). At discharge, patients were tested with the modified Rankin scale in order to assess level of disability and dependence in daily activities. After 12–14 months post ictus, patients were seen for a follow-up visit, when a battery of tests was administered (Hospital Anxiety and Depression Scale (HADS), Brief COPE Inventory, Utrecht Scale for Evaluation of Rehabilitation–Participation (US-ER-P), the Montreal Cognitive Assessment (MoCA)). All instruments were administered face-to-face by trained researchers. Questionnaires were completed in paper form and checked for missing responses before data entry.

Tests used:

The Hunt and Hess (HH) scale is a five-grade clinical tool used to assess the initial severity of aneurysmal subarachnoid hemorrhage. Grades range from I (minimal symptoms) to V (deep coma and severe neurological impairment) [20]. The modified Rankin Scale (mRS) is a widely used measure of global disability after neurological injury, ranging from 0 (no symptoms) to 6 (death). It evaluates the degree of functional independence in daily activities [21].

Psychological distress was assessed using the Hospital Anxiety and Depression Scale (HADS) [22], which consists of 14 items rated on a 4-point scale (0–3). It includes two 7- item subscales for anxiety (HADS-A) and depression (HADS-D), with each subscale ranging from 0 to 21. Higher scores indicate greater symptom severity. Clinically relevant symptoms were defined as scores ≥8 in both categories.

Coping strategies were assessed using the 28-item Brief COPE Inventory [23]. Fol-lowing commonly used higher-order models in the literature, the subscales were grouped into three domains: active coping (active coping, planning, positive reframing, instrumental support), emotional coping (emotional support, venting, humor, religion, acceptance), and avoidant coping (self-distraction, denial, behavioral disengagement, substance use, self-blame). Higher scores indicate greater use of the respective coping style.

Participation was evaluated using the Utrecht Scale for Evaluation of Rehabilitation–Participation (USER-P) [24], a 31-item self-report instrument that provides three domain scores: frequency of participation, participation restrictions, and satisfaction with participation. The scale assesses involvement in everyday and social activities (e.g., work, household tasks, mobility, leisure, and social interactions), as well as perceived limitations and satisfaction with performing these activities. Scores are transformed to a 0–100 scale, with higher values indicating better outcomes (greater participation frequency, fewer re-strictions, and higher satisfaction).

Cognition was assessed with the Montreal Cognitive Assessment (MoCA) [25], a 30-point screening tool evaluating attention, memory, executive functions, language, visuospatial ability, and orientation. Scores ≤ 22 were considered indicative of cognitive impairment [26]. Patients were classified as cognitively impaired (≤ 22) or preserved (≥ 22) for subgroup analyses.

### 2.4. Statistical Analysis

Descriptive statistics summarized demographic, clinical, cognitive, and participation data. Group differences in continuous variables (MoCA, Brief-COPE, USER-P) were tested with independent-samples *t*-tests or Mann–Whitney U tests, and categorical variables (e.g., MoCA ≤ 22) with Fisher’s exact test. Within the patient group, subgroup analyses compared USER-P outcomes between cognitively impaired (MoCA ≤ 22) and preserved (MoCA > 22) patients. Pearson correlations examined associations between MoCA and USER-P domains. Multiple linear regressions identified independent predictors of each USER-P domain (restrictions, frequency, satisfaction). Effect sizes were calculated using Cohen’s d for between-group comparisons. Analyses were conducted in IBM SPSS Statistics v21.0, with *p* < 0.05 considered significant.

## 3. Results

Forty patients who completed the one-year follow-up and seventy-five healthy controls were included in the final analysis. Controls were individually matched to patients by sex, age, and education level. The patient group comprised 26 women (65.0%) with a mean age at assessment of 53.8 years (SD = 9.8; range: 35–74 years). At admission, 11 patients (27.5%) had Hunt and Hess grade I and 29 patients (72.5%) had grade II. Thirty-five patients were matched with two healthy controls each, and five with one. Premorbid comorbidities, including hypertension, cardiovascular disease, hyperlipidemia, and diabetes, were significantly more prevalent in the patient group compared to controls (80.0% vs. 52.5%, *p* = 0.006). Detailed demographic and clinical characteristics are summarized in Table 1. There were no significant differences in age, sex, Hunt and Hess grade, or aneurysm location between patients who completed the one-year follow-up and those lost to follow-up (all *p* > 0.05).

Group differences in psychological distress, coping strategies, and participation outcomes are presented in Table 2. There was no statistically significant difference in mean HADS depression scores between patients and controls (*p* = 0.194; d = 0.21). For HADS anxiety, patients had higher mean scores, but the difference did not reach statistical significance (*p* = 0.079; d = 0.37). However, a significantly greater proportion of patients scored above both clinical cutoffs for anxiety (≥8: 40.0% vs. 18.7%, *p* = 0.024; ≥11: 20.0% vs. 2.7%, *p* = 0.003), and a higher proportion of patients reached the severe depression cutoff (≥11: 10.0% vs. 0%, *p* = 0.013). Cognitive outcomes differed markedly between groups. Patients had significantly lower MoCA scores compared to controls (22.3 ± 5.3 vs. 27.2 ± 2.2, *p* < 0.001; d = 1.1), with 42.5% of patients scoring ≤ 22, consistent with cognitive impairment, compared to only 2.7% of controls (*p* < 0.001). Exploratory domain-level analyses of the MoCA revealed significantly lower scores in visuospatial/executive functions, orientation, delayed recall, and naming/language among patients compared with controls (all *p* < 0.01 after Holm–Bonferroni correction). Attention and abstraction showed smaller, non-significant differences. When stratifying patients by cognitive status (MoCA ≤ 22 vs. > 22), those with cognitive impairment had lower participation frequency (*p* = 0.057) and satisfaction (*p* = 0.070), and significantly greater participation restrictions (*p* = 0.014). Anxiety scores were also significantly higher in the cognitively impaired subgroup (*p* = 0.048), whereas depression scores did not differ significantly (*p* = 0.21).

**Figure 2:**
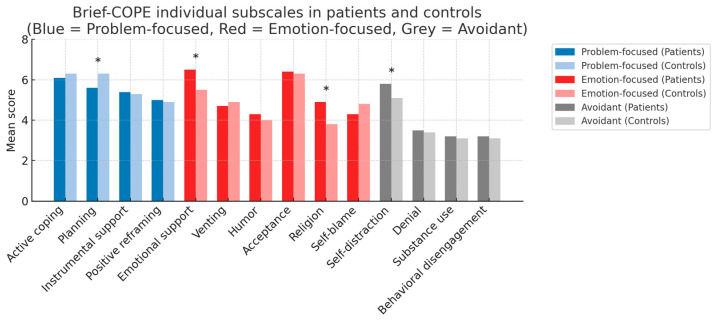
Mean Brief-COPE item scores in patients and healthy controls. Bar colors indicate coping strategies within the coping styles Problem-Focused (blue), Emotion-Focused (red), and Avoidant (gray). Darker shades represent patients (aSAH group), lighter shades represent healthy controls. * *p* < 0.05.

Regarding coping strategies, there were no significant differences between groups in active or emotional coping (d = 0.39 for emotional coping). However, patients reported significantly higher use of avoidant coping strategies than controls (*p* = 0.048; d = 0.16). At the level of individual BRIEF-COPE subscales, patients used self-distraction (*p* = 0.040), emotional support (*p* = 0.002), and religion (*p* = 0.002) more often than controls, whereas planning was significantly more common among controls (*p* = 0.010). There was also a trend toward higher denial in patients (*p* = 0.095), but this did not reach statistical significance. No significant differences were found for other coping subscales. These findings are illustrated in Figure 2, which presents mean scores for each Brief-COPE subscale and their grouping into the three coping domains (problem-focused, emotion-focused, and avoidant).

Participation outcomes (USER-P) were significantly poorer in patients across all domains. Patients reported lower frequency of participation (median [IQR]: 35 [28–51] vs. 51 [43–57]; *p* < 0.001; Cohen’s *d* = −1.22), greater participation restrictions (82 [58–92] vs. 100 [97–100]; *p* < 0.001; *d* = −1.21), and lower satisfaction with participation (65 [49–75] vs. 75 [70–85]; *p* < 0.001; *d* = −0.69) compared to controls.

The distribution of participation restrictions across specific activities one year after aSAH is shown in Table 3. Percentages are calculated based on valid responses; ‘not applicable’ items were excluded. The highest rates of reported restrictions were observed in sports or other physical exercise (54%) and in paid/unpaid work or education (53%). Substantial limitations were also seen in household duties (44%), outdoor mobility (40%), leisure activities at home (36%), and day trips or other outdoor activities (34%). Social participation (e.g., visiting friends or family [26%], receiving visitors [15%], contacting others by phone or computer [18%]) was less commonly restricted. These findings indicate that patients most frequently experienced restrictions in physically demanding and routine functional activities.

To further explore the impact of cognitive impairment on participation outcomes, patients were stratified according to MoCA score (≤22 vs. >22). Descriptive statistics for USER-P domains in these subgroups are presented in Table 4. When stratifying patients by cognitive status, those with cognitive impairment (MoCA ≤ 22) showed lower participation frequency (*p* = 0.057) and satisfaction (*p* = 0.070), and significantly greater participation restrictions (*p* = 0.014) compared with cognitively preserved patients.

Pearson correlations showed that higher HADS anxiety and depression scores were strongly associated with greater participation restrictions (r = −0.65 and r = −0.61, both *p* < 0.001) and lower satisfaction (r = −0.59 and r = −0.58, both *p* < 0.001). Avoidant coping was also correlated with increased restrictions (r = −0.42, *p* = 0.007) and lower satisfaction (r = −0.40, *p* = 0.011). MoCA scores were positively associated with participation frequency (r = 0.32, *p* = 0.045), fewer restrictions (r = 0.51, *p* = 0.001), and higher satisfaction (r = 0.43, *p* = 0.005). Restrictions and satisfaction were strongly correlated (r = 0.82, *p* < 0.001). Older age and employment were linked to fewer restrictions and higher satisfaction, while higher education was related to greater satisfaction.

Multivariate regression analyses (Table 5) showed that higher anxiety, higher depression, and greater use of avoidant coping strategies were all independently associated with greater participation restrictions at one year after aSAH (β = −0.33, *p* = 0.015; β = −0.31, *p* = 0.019; β = −0.27, *p* = 0.042; adjusted R^2^ = 0.48). None of the demographic variables remained statistically significant predictors in the final model for restrictions. For participation frequency, lower MoCA scores were significantly associated with poorer outcomes (β = 0.32, *p* = 0.045), while employment status and anxiety showed trends toward significance (*p* = 0.053 and *p* = 0.072, respectively; adjusted R^2^ = 0.21). Satisfaction with participation was independently predicted by lower depressive symptoms (β = −0.35, *p* = 0.011), less avoidant coping (β = −0.29, *p* = 0.034), and being employed (β = 0.41, *p* = 0.002; adjusted R^2^ = 0.52). No other demographic or clinical variables were significant predictors for any USER-P outcomes in these models. Spearman correlations showed no significant associations between premorbid status and participation outcomes (r = −0.11 to −0.21, all *p* > 0.10). When tested alone, premorbid status showed a modest negative association with satisfaction (β = −0.41, *p* = 0.025, adjusted R^2^ = 0.13); however, this effect was no longer significant when psychological, cognitive, and employment-related factors were added to the regression model (β = −0.18, *p* = 0.42).

## 4. Discussion

Our findings show that, even one year after aSAH, patients experience substantial limitations in participation and considerable psychological distress, despite having initially mild hemorrhage severity and good neurological recovery. This highlights that recovery extends beyond neurological recovery and underscores the importance of psychosocial outcomes as primary endpoints after aSAH. Compared to matched controls, patients reported significantly lower frequency of engagement, greater restrictions, and reduced satisfaction in many activities, particularly in physically demanding domains such as work and sports, particularly in physically demanding domains such as work and sports, although social and leisure activities were also affected.

Symptoms of depression and anxiety are commonly present in patients after aSAH. Tang et al. [7] found that about 30% of patients develop depression with a chronic course, while Kreiter et al. [8] reported depressive symptoms in up to half of patients in the first year. Persson et al. [26] documented persistently high anxiety rates even seven years post-ictus. In our study, mean HADS scores did not differ significantly between groups, but clinically relevant symptoms were more frequent among patients (32% anxiety, 23% depression), consistent with Ackermark et al. [9]. Markedly higher proportion of patients exceeding clinical cut-offs underlines that group averages may underestimate the true burden of psychological distress in this population. These findings underscore the need to move beyond group averages and highlight the importance of long-term psychological follow-up.

In our sample, avoidant coping was not only more frequent but also independently predicted poorer outcomes, underscoring its relevance as a target for rehabilitation. Although the between-group effect size for avoidant coping was small (d = 0.16), its clinical relevance became evident within the patient group, where greater use of avoidance was strongly associated with higher participation restrictions and lower satisfaction. This suggests that even modest differences at the group level may translate into substantial negative consequences for individuals relying on maladaptive strategies. This finding extends previous data [17,27] by showing that the negative impact of avoidance is already evident one year after aSAH. Longitudinal studies also identified passive coping as a predictor of persistent emotional problems [9], while more recent evidence points to its association with fatigue, with acceptance emerging as protective [18]. Qualitative reports similarly describe withdrawal and concealment of symptoms despite valuing social support [16]. Together, these findings indicate that avoidant coping should be considered an independent behavioral target for rehabilitation.

Beyond physical and cognitive recovery, reintegration into meaningful personal, social, and professional roles remains a fundamental rehabilitation goal. Participation captures not only functional independence but also autonomy and fulfillment. Although longitudinal research shows gradual improvement over time [14], many patients continue to face limitations even years after the event [19]. These are not explained by physical status alone, as subjective cognitive complaints and psychological symptoms are among the strongest predictors of restrictions [14,19].

Our data confirm that participation restrictions remain a major challenge one year after aSAH, particularly in demanding domains such as work, household responsibilities, and sports. More than half of our patients reported difficulties in these areas, which is consistent with earlier studies [5,13,28,29]. One possible explanation is that patients are commonly advised to avoid strenuous physical exertion and sports for up to one year after the hemorrhage, which may contribute to lower engagement and greater perceived limitations in these domains. In addition, post-aSAH fatigue, frequently described even in patients with good neurological recovery, may further reduce tolerance for physically demanding activities, reinforcing avoidance patterns and amplifying participation restrictions [30]. Importantly, we found that psychological distress, maladaptive coping, and lower MoCA scores, rather than demographic or clinical factors, were most strongly linked to reduced participation. The discrepancy between the correlation analyses and the multivariable regression models likely reflects shared variance between cognitive functioning and psychological distress. Although MoCA scores correlated with all three USER-P domains, their independent predictive value diminished once anxiety, depression, and avoidant coping were entered into the model—psychological variables that more directly shape subjective experiences of functioning. This pattern is consistent with previous findings showing that subjective cognitive difficulties and emotional distress are more predictive of participation outcomes than objective cognitive performance [12,30]. Consequently, MoCA remained a significant predictor only of participation frequency, the domain most dependent on cognitive efficiency, planning, and executive control. In contrast, restrictions and satisfaction reflect perceptual and evaluative aspects of participation and are therefore more strongly influenced by emotional distress and maladaptive coping. Our subgroup analysis further demonstrated that patients with MoCA ≤ 22 had markedly greater participation restrictions and lower satisfaction compared with cognitively preserved patients, with medium-to-large effect sizes. This indicates that cognitive impairment remains clinically meaningful for real-world functioning, even if it does not independently predict all USER-P domains in multivariable models. Its impact appears most evident in tasks that require planning, initiation, and cognitive efficiency, whereas perceived restrictions and satisfaction are more strongly shaped by emotional distress and coping. Together, these results support previous evidence that psychological and cognitive factors outweigh neurological severity in predicting participation outcomes [14,17,27].

Neither sex, marital status, nor clinical characteristics were associated with participation outcomes. Premorbid status also showed no independent association with any USER-P domain after adjustment, indicating that pre-existing health conditions do not meaningfully shape long-term participation when psychological and cognitive factors are considered. Employment status emerged as the only demographic factor linked to higher satisfaction and fewer restrictions, consistent with previous findings on the importance of occupational and social engagement [17,19].

Future studies should clarify the longitudinal course of psychological distress, cognitive impairment, and participation outcomes in aSAH survivors, and investigate how these factors interact. Such work will be essential for developing targeted rehabilitation strategies that address psychological symptoms, coping, and cognition to improve long-term psychosocial recovery.

## 5. Limitations

This study has several limitations. The relatively small sample size may limit generalizability, particularly for subgroup comparisons; however, the observed effect sizes suggest that these findings are clinically meaningful. Outcomes were assessed only at the one-year follow-up, which prevents conclusions about longitudinal trajectories or causal relationships. The sample consisted exclusively of patients with good neurological recovery from a single center, which may not fully represent the broader population of aSAH survivors. Initial clinical severity was determined solely with the Hunt and Hess (HH) scale, as this was the only grading system consistently documented in routine practice. Alternative scales such as the WFNS or Fisher grades were not systematically available, which may limit comparability with some studies. Nevertheless, restricting our sample to patients with HH grade I or II minimized the confounding influence of severe neurological deficits on long-term psychosocial outcomes. Cognitive functioning was assessed using the MoCA, which provides a brief global screening across several domains but does not allow for detailed neuropsychological characterization. Although we performed exploratory domain-level analyses to complement the global score, the use of a single screening tool may not fully capture the breadth of domain-specific impairments known to occur after aSAH. Finally, psychological distress, coping strategies, and participation outcomes were measured exclusively with self-report instruments (HADS, Brief COPE, USER-P). Although widely validated and commonly used in this population, self-report measures may be subject to bias, and the absence of clinician-rated assessments (e.g., Hamilton scales) limits the objectivity of some findings.

## 6. Conclusions

Supporting long-term participation after aSAH requires more than neurological recovery or independence in basic activities of daily living. Comprehensive, multidisciplinary rehabilitation that addresses psychological well-being, cognitive functioning, and coping strategies is essential. Routine cognitive screening and targeted interventions should be integrated into long-term follow-up after aSAH to improve participation and psychosocial outcomes. By directly addressing these factors, clinicians might better support survivors in regaining autonomy and fulfilling meaningful social and professional roles. Our findings highlight that successful participation depends not only on physical recovery but also on the effective management of psychological and cognitive challenges.

## Figures and Tables

**Table 1:** Demographic, clinical, and psychological characteristics of the study sample.

	Patients (*n* = 40)	Controls (*n* = 75)
Sex, *n* (%) female	26 (65.0%)	53 (66.3%)
Sex, *n* (%) male	14 (35.0%)	27 (33.7%)
Age at test, mean (SD)	53.8 (9.8)	53.1 (9.6)
Education: low/intermediate	36 (90.0%)	68 (90.7%)
Education: high	4 (10.0%)	7 (9.3%)
Marital status: Married	23 (57.5%)	50 (62.5%)
Divorced	2 (5.0%)	10 (12.5%)
Widowed	8 (20.0%)	5 (6.2%)
Single	7 (17.5%)	10 (12.5%)
Living with others	37 (92.5%)	68 (85.0%)
Living alone	3 (7.5%)	7 (8.8%)
Employed	18 (45.0%)	56 (70.0%)
Unemployed	9 (22.5%)	4 (5.0%)
Retired	13 (32.5%)	15 (18.8%)
Premorbid comorbidities:		
Hypertension	25 (62.5%)	31 (38.8%)
Cardiovascular disease	4 (10.0%)	6 (7.5%)
Hyperlipidemia	7 (17.5%)	9 (11.2%)
Diabetes	6 (15.0%)	2 (2.5%)
At least one comorbidity	32 (80.0%)	42 (52.5%)
Anterior circulation aneurysm	37 (92.5%)	—
Posterior circulation aneurysm	3 (7.5%)	—
HH grade I	11 (27.5%)	—
HH grade II	29 (72.5%)	—
Length of hospital stay (LOS), mean (SD)	12.8 (5.9)	—
Rebleeding	0 (0.0%)	—
Ischemia	9 (22.5%)	—
Hydrocephalus	2 (5.0%)	—
HADS—depression, mean (SD)	5.0 (4.0)	3.6 (2.8)
HADS—depression ≥ 8, *n* (%)	9 (22.5%)	1 (1.3%)
HADS—depression ≥ 11, *n* (%)	4 (10.0%)	0 (0.0%)
HADS—anxiety, mean (SD)	7.1 (4.5)	5.0 (2.9)
HADS—anxiety ≥ 8, *n* (%)	14 (35.0%)	11 (13.8%)
HADS—anxiety ≥ 11, *n* (%)	8 (20.0%)	2 (2.5%)
MoCA, mean (SD)	22.3 ± 5.3	27.2 ± 2.2
MoCA ≤ 22, *n* (%)	17 (42.5%)	2 (2.7%)

Abbreviations: HH = Hunt and Hess grade; LOS = Length of hospital stay; HADS = Hospital Anxiety and Depression Scale; MoCA = Montreal Cognitive Assessment; SD = Standard deviation; ≥ = equal or greater than; — = not applicable.

**Table 2:** Mood, coping, cognition and participation outcomes in patients vs. controls.

Scale	Patients (*n* = 40)	Controls (*n* = 75)	*p*-Value
HADS Anxiety	7.1 (4.5)	5.0 (2.9)	0.079
≥8, *n* (%)	16 (40.0%)	14 (18.7%)	0.024
≥11, *n* (%)	8 (20.0%)	2 (2.7%)	0.003
HADS Depression	5.1 (4.0)	3.6 (2.8)	0.194
≥8, *n* (%)	10 (25.0%)	8 (10.7%)	0.081
≥11, *n* (%)	4 (10.0%)	0 (0.0%)	0.013
Active coping	21.95 (4.22)	22.93 (4.71)	0.242
Emotional coping	30.55 (5.58)	28.85 (5.66)	0.177
Avoidant coping	16.12 (3.76)	14.72 (3.97)	0.048
MoCA	22.3 ± 5.3	27.2 ± 2.2	<0.001
USER-P Frequency	36.86 (14.14) 35 (28–51)	49.92 (10.65) 51 (43–57)	<0.001
USER-P Restrictions	72.88 (26.07) 82 (58–92)	96.28 (7.74) 100 (97–100)	<0.001
USER-P Satisfaction	62.00 (19.61) 65 (49–75)	75.53 (13.08) 75 (70–85)	<0.001

Legend: HADS = Hospital Anxiety and Depression Scale; ≥8, *n* (%)/≥11, *n* (%): number (percentage) of participants above HADS cut-offs, compared by Fisher’s exact test. Active coping, emotional coping, avoidant coping = subscales of the Brief COPE inventory. MoCA = Montreal Cognitive Assessment. USER-P = Utrecht Scale for Evaluation of Rehabilitation–Participation. Frequency = frequency of participation. Restrictions = participation restrictions. Satisfaction = satisfaction with participation. Mean (SD) = arithmetic mean (standard deviation), compared by *t*-test. Median (IQR) = median (interquartile range), compared by Mann–Whitney U test. *p* < 0.05 considered significant.

**Table 3:** Participation restrictions by activity (USER-P, 1 year post-aSAH).

Activity	*n* with Restriction	% with Restriction	*n* Valid
Paid work, unpaid work or education	16	53%	30
Household duties	17	44%	39
Outdoor mobility	16	40%	40
Sports or other physical exercise	20	54%	37
Going out	10	30%	33
Day trips and other outdoor activities	13	34%	38
Leisure activities at home	14	36%	39
Relationship with your partner	10	31%	32
Going to visit family or friends	10	26%	39
Family or friends coming to visit	6	15%	40
Contacting others by phone or computer	7	18%	39

**Table 4:** Participation outcomes by cognitive status (MoCA ≤ 22 vs. >22) in the patient group.

	MoCA > 22 (*n* = 23)	MoCA ≤ 22 (*n* = 17)	*p*-Value	Effect Size
Participation frequency	40.47 ± 14.12	31.98 ± 13.00	0.057	*d* = 0.62
Participation restrictions	81.69 ± 21.24	60.96 ± 27.81	0.014	r = 0.39
Participation satisfaction	67.07 ± 16.25	55.15 ± 22.09	0.070	*d* = 0.63

Legend: MoCA—Montreal Cognitive Assessment; SD—standard deviation; *d*—Cohen’s d; r—effect size.

**Table 5:** Multivariate regression models for USER-P participation outcomes one year after aSAH.

Outcome	Predictor	Standardized β	*p*-Value	Adjusted R^2^
Restrictions Score	HADS Anxiety Skor	−0.33	0.015	0.48
	HADS Depression Skor	−0.31	0.019	
	Avoidant coping	−0.27	0.042	
	Employment	0.21	0.093	
	Age	−0.18	0.172	
	Education	0.16	0.198	
	MoCA	0.09	0.28	
	Premorbid status	−0.12	0.47	
Frequency Score	HADS Anxiety Skor	−0.24	0.072	0.21
	Employment	0.28	0.053	
	Age	−0.19	0.166	
	Avoidant coping	−0.15	0.202	
	HADS Depression Skor	−0.14	0.225	
	Education	0.13	0.229	
	MoCA	0.32	0.045	
	Premorbid status	−0.09	0.51	
Satisfaction Score	Employment	0.41	0.002	0.52
	HADS Depression Skor	−0.35	0.011	
	Avoidant coping	−0.29	0.034	
	HADS Anxiety Skor	−0.12	0.263	
	Age	−0.10	0.297	
	Education	0.11	0.282	
	MoCA	0.11	0.22	
	Premorbid status	−0.18	0.42	

Legend: Standardized regression coefficient (β) represents the strength and direction of the association between each predictor and the outcome variable. Adjusted R^2^ indicates the proportion of variance in the outcome variable explained by the model, adjusted for the number of predictors. USER-P = Utrecht Scale for Evaluation of Rehabilitation–Participation (restrictions, frequency, satisfaction); HADS-A/HADS-D = Hospital Anxiety and Depression Scale, anxiety and depression subscales; Avoidant coping = avoidant coping score from the Brief COPE questionnaire; MoCA = Montreal Cognitive Assessment.

## Data Availability

The datasets generated and analyzed during the current study are available from the corresponding author upon reasonable request.
